# Development of a Rapid Insulin Assay by Homogenous Time-Resolved Fluorescence

**DOI:** 10.1371/journal.pone.0148684

**Published:** 2016-02-05

**Authors:** Zachary J. Farino, Travis J. Morgenstern, Julie Vallaghe, Nathalie Gregor, Prashant Donthamsetti, Paul E. Harris, Nicolas Pierre, Robin Freyberg, Fabienne Charrier-Savournin, Jonathan A. Javitch, Zachary Freyberg

**Affiliations:** 1 Department of Psychiatry, College of Physicians & Surgeons, Columbia University, New York, New York, United States of America; 2 Division of Molecular Therapeutics, New York State Psychiatric Institute, New York, New York, United States of America; 3 Research Department, Cisbio Bioassays, Codolet, France; 4 Department of Pharmacology, College of Physicians & Surgeons, Columbia University, New York, New York, United States of America; 5 Division of Endocrinology, Department of Medicine, College of Physicians & Surgeons, Columbia University, New York, New York, United States of America; 6 Department of Psychology, Stern College for Women, Yeshiva University, New York, New York, United States of America; Martin Luther University, GERMANY

## Abstract

Direct measurement of insulin is critical for basic and clinical studies of insulin secretion. However, current methods are expensive and time-consuming. We developed an insulin assay based on homogenous time-resolved fluorescence that is significantly more rapid and cost-effective than current commonly used approaches. This assay was applied effectively to an insulin secreting cell line, INS-1E cells, as well as pancreatic islets, allowing us to validate the assay by elucidating mechanisms by which dopamine regulates insulin release. We found that dopamine functioned as a significant negative modulator of glucose-stimulated insulin secretion. Further, we showed that bromocriptine, a known dopamine D2/D3 receptor agonist and newly approved drug used for treatment of type II diabetes mellitus, also decreased glucose-stimulated insulin secretion in islets to levels comparable to those caused by dopamine treatment.

## Introduction

Insulin is an anabolic hormone that regulates metabolism and energy homeostasis. The release of insulin by pancreatic beta cells in response to increases in extracellular glucose promotes glucose uptake in insulin-sensitive tissues [1]. Disruption of the regulation of insulin secretion leads to profound global metabolic effects that can result in diabetes mellitus and tissue damage [2]. Many aspects of insulin release, including the mechanisms regulating glucose-stimulated insulin secretion (GSIS), require further study. For example, emerging evidence suggests that peripheral dopamine (DA) is an important modulator of GSIS [3]. Moreover, several antipsychotic drugs, which target DA D_2_-like receptors including D_2_ (D2R) and D_3_ (D3R) receptors, significantly dysregulate insulin secretion [3,4]. Thus, a rapid, cost-effective and scalable assay for quantitating insulin levels would facilitate further studies of GSIS and drug-induced metabolic syndromes and, more generally, would be useful in a variety of clinical, academic and industrial settings.

To date, the predominant methods used to measure insulin are radioimmunoassay (RIA) and enzyme-linked immunosorbent assay (ELISA) [5–7]. RIA was the first widely used assay for insulin detection [5]. However, this approach is limited by potential safety concerns due to the use of radiolabeled antigen, the instability of the reagents and the need for extended incubation and washing steps [8]. The development of an ELISA-based assay has allowed for the detection of insulin without the need for radioactive reagents. Although ELISA is currently the gold standard assay for measuring insulin, it is expensive (>$2/sample), and, like RIA, is also labor intensive and thus relatively low-throughput [9–11]. The recently developed homogenous insulin assay, AlphaLISA, relies on oxygen channeling chemistry to generate singlet oxygen which initiates a chemiluminescent reaction following insulin binding [11]. This approach, which requires fewer overall steps compared to ELISA, has facilitated higher-throughput screening. Nevertheless, this assay displays limited signal stability, as it is highly sensitive to ambient light exposure, singlet oxygen sequestration and photobleaching [12]. Thus, a real need still exists for a reliable, rapid and affordable insulin assay amenable to high-throughput studies. To address this, here we used homogenous time-resolved fluorescence (HTRF) [13,14] to design a cell-based assay for rapid detection and measurement of insulin, resulting in a simple yet robust, cost-effective ($0.20/sample) and sensitive insulin detection assay capable of being read by numerous microplate readers. We then used this assay to measure insulin release from two complementary experimental systems: INS-1E cells, a widely used and well-characterized insulin-secreting rat beta cell-derived cell line [15] as well as from mouse pancreatic islets. As a proof-of-principle, we further validated our HTRF insulin assay by examining the roles of DA and D2R/D3R signaling in mediating GSIS, which we and colleagues have recently shown to act as components of an autocrine/paracrine negative feedback mechanism [3,16,17]. Lastly, we expanded on these findings by examining effects of bromocriptine, a known dopamine D2/D3 receptor agonist [18,19], on GSIS in mouse islets using our HTRF-based assay. Though bromocriptine was recently newly approved for treatment of type II diabetes mellitus [18,20–23], to date, the precise molecular mechanisms responsible for its efficacy remain poorly understood. Here, consistent with earlier data suggesting that the drug can modify GSIS [24], we show that bromocriptine acts directly on islets as a negative mediator of GSIS, providing a putative molecular mechanism for its actions in the pancreas.

## Materials and Methods

### Cell Culture

Rat beta cell-derived INS-1E cells (gift of P. Maechler, Université de Genève; [15]) were maintained in a humidified 37°C incubator with 5% CO_2_. The cells were cultured with RPMI 1640 medium (Life Technologies, Norwalk, CT) supplemented with 5% (v/v) heat inactivated fetal bovine serum, 2 mM L-glutamine, 10 mM HEPES, 1 mM sodium pyruvate, 100 units/mL penicillin, 100 μg/mL streptomycin, and 50 μM 2-mercaptoethanol.

### Mice and Pancreatic Islet Preparation

All animals were housed and handled in accordance with all appropriate NIH guidelines through the Columbia University Institute of Comparative Medicine. The institutional review board and ethics committee of Columbia University Medical Center approved the study. We abided by all appropriate animal care guidelines including ARRIVE guidelines for reporting animal research (S1 File) [25,26]. All efforts were made to ameliorate animal suffering. Animal sacrifice was humanely performed by cervical dislocation for adult mice.

Wildtype C57Bl6/J mice were purchased from rotavirus-free colonies of Jackson Laboratory (Bar Harbor, ME). For mouse pancreatic islet preparations, wildtype 8–10 week old male and female C57Bl6/J mice weighing ~25–30 g were used. Mice were housed in cages with a 12:12 light:dark cycle and had access to food and water *ad lib* at all times. Pancreatic islets were isolated via collagenase digestion as described previously [27]. Each experiment used 3 mice to obtain sufficient numbers of islets for that respective day’s conditions with every condition performed either in triplicate or quadruplicate (21 mice used in total). Islets were seeded at the following densities: 10 islets per well in 24-well plates or single islets in each well of a 96-well plate. We then standardized the islets per well based on islet size and morphology using a dissecting microscope according to methods established in earlier studies [28–31]. Islets of relatively uniform size and shape were evenly distributed throughout the wells, as indicated by Hopcroft *et al*. (1985) and Colella *et al*. (1985) [29,30]. The islets were then cultured free-floating overnight in RPMI 1640 media supplemented with 10% fetal bovine serum prior to experimental use the following day.

### Compounds

The compounds used in the present study were as follows and purchased from Sigma-Aldrich (St. Louis, MO) unless indicated otherwise: D-glucose, dopamine (3-hydroxytyramine HCl), HEPES, sodium pyruvate, penicillin, streptomycin, 2-mercaptoethanol. Human, bovine and porcine insulin were also obtained from Sigma-Aldrich. Rodent (rat/mouse-reactive) insulin was obtained from ALPCO (Salem, NH). Human proinsulin was obtained from R&D Systems (Minneapolis, MN) and human C-peptide was purchased from AnaSpec, EGT (Fremont, CA). Bromocriptine mesylate was purchased from Tocris (Bristol, United Kingdom).

### HTRF insulin assay

The HTRF insulin detection assays were performed in either half-area, 96-well or 384-well plates (Greiner Bio-One, Monroe, NC) by adding both anti-insulin antibody coupled to Europium cryptate and a second anti-insulin antibody coupled to XL665 (Cisbio Bioassays, Bedford, MA) to the respective samples in a 1:1 ratio (total antibody volume:sample volume). Antibody incubation was conducted for 2 h at room temperature (25°C) with pH 7 buffer unless otherwise specified. The resulting fluorescence emissions were read by a multi-mode microplate reader (PHERAstar FS, BMG Labtech, Ortenberg, Germany) which utilized a 337 nm nitrogen laser for fluorophore excitation, a 620 nm filter for Europium cryptate fluorescence reading, and a 665 nm filter for the XL665 fluorescence detection. Data were reported as the ratio of fluorescence measured at 665nm (XL665) and 620 nm (Europium cryptate) with the signal at 620 nm functioning as an internal standard following a 40 μs time delay. Insulin standards were set up for each plate by diluting known insulin concentrations (0.312–15 ng/mL). HTRF measurements of these standards were fit to a standard curve fit with a second order polynomial (quadratic) regression line (y = B_0_+B_1_x+B_2_x^2^) via GraphPad Prism software (version 6.0, GraphPad Software, Inc., La Jolla, CA) allowing for interpolation of raw HTRF values from the experimental samples to known insulin concentration values.

The ability of the assay’s antibodies to recognize insulin across species involved measurement of HTRF signals of increasing concentrations of human, bovine, porcine and rodent insulin. The respective HTRF signals were plotted as Log [insulin concentration] versus the ΔF% where ΔF% was defined as [(mean HTRF ratio of a sample)–(mean HTRF ratio of the zero standard)/(mean HTRF ratio of the zero standard)]; the zero standard was defined as a sample with assay buffer only (0 ng/mL insulin). This permitted comparison of HTRF signals from the respective species’ insulin across a range of insulin concentrations (0.01–10 nM). Additionally, we measured the ability of the assay’s antibodies to recognize either human proinsulin or human insulin C-peptide. In the case of the proinsulin recognition, we measured the respective HTRF signals across a range of proinsulin concentrations (12.3–195 pM) and compared with equivalent concentrations of human insulin. We also measured HTRF signals for C-peptide across a broad concentration range (2–20,000 pM).

For cell-based glucose stimulation experiments, we measured the amount of insulin secreted in collected supernatant as well as residual cellular insulin content from whole cell lysate. INS-1E cells were seeded into individual wells of a 24-well plate at an initial seeding density of 5.0 x 10^5^ cells/well unless described otherwise. RPMI 1640 media was exchanged 24 h after cell seeding and experiments were conducted the following day. On the day of the insulin secretion assay, the INS-1E cells underwent a glucose starvation step (1 h, 37°C) by exchange of RPMI 1640 medium (which contains 11 mM glucose) with glucose-free (0 mM glucose) KRB buffer (132.2 mM NaCl, 3.6mM KCl, 5mM NaHCO_3_, 0.5 mM NaH_2_PO_4_, 0.5 mM MgCl_2_, 1.5 mM CaCl_2_, and 0.001 g/mL bovine serum albumin), as described previously [15]. The cells were then stimulated with glucose (20 mM glucose unless specified otherwise) in KRB buffer for 90 min, 37°C; 20 μL of supernatant from each sample was subsequently collected for the insulin assay. Similarly, for mouse islets secretion studies, islets seeded in either 24-well or 96-well plates (for single islet secretion experiments) were glucose-starved in KRB buffer containing 2.8 mM glucose (1 h, 37°C) as described in earlier studies [3,32]. Following starvation, the islets were stimulated with 20 mM glucose in KRB in the presence or absence of additional drugs. At the conclusion of stimulation, we collected 10 μL of supernatant from each sample to measure secreted insulin content. For the INS-1E-cell-based assay (conducted in the 96-well plates), 10 μL of each antibody (20 μL total antibody volume) was added to 20 μL of cell supernatant. Likewise, in the islet-based assays (conducted in 384-well plates), 5 μL of each antibody was added to 10 μL of islet supernatant. Following 30–90 min of glucose stimulation of the INS-1E cells, HTRF signal from the supernatant was ultimately reported either as insulin concentration values (in ng/mL) or as the percent of maximal stimulated insulin secretion which was calculated as the ratio of [insulin concentration for a respective sample]/[maximal insulin concentration within an experiment]. Further, GSIS was defined as the difference between maximal stimulated and basal (unstimulated) insulin release with effects of drugs on GSIS determined relative to this difference; the unstimulated condition was defined as 0 mM glucose for experiments using INS-1E cells and 2.8 mM glucose for pancreatic islet studies of GSIS as based on earlier studies [3,15,32].

To measure intracellular insulin content in INS-1E cells, we first washed adherent INS-1E cells with KRB buffer to remove any secreted insulin from the supernatant. We next added a Triton X-100-based cell lysis solution consisting of 25% (v/v) 4x concentrated cell lysis buffer (4X Cell phospho/total protein lysis buffer #1 containing 25% Triton X-100, pH 7; Cisbio Bioassays, Bedford, MA) and 75% KRB (v/v) to the cells. The cells were then shaken at 25°C for 30 min. Insulin content of the prepared lysates was subsequently determined via HTRF. Similarly, to assay intracellular insulin content in pancreatic islets, islets from wildtype C57Bl6/J mice were isolated (as described earlier) and cultured overnight in RPMI 1640 supplemented with 10% newborn calf serum overnight (5% CO_2_, 37°C). Islets were then lysed after treatment with 25% (v/v) 4x Cell phospho/total protein lysis buffer #1 and 75% KRB (v/v) at 25°C for 30 min. The resulting islet lysate was used to determine intra-islet insulin concentration by HTRF.

Given that insulin concentrations in cell and islet-derived supernatants and lysates were typically greater than the concentrations used to generate the standard curves, to determine accurate insulin concentrations, we needed to bring our samples’ insulin concentrations into the standard curve’s linear range by appropriately diluting our samples. Following interpolation of the diluted insulin concentrations, we obtained the insulin concentrations of the original, undiluted samples by multiplying the diluted insulin concentrations by the respective dilution factors for each treatment condition.

### ELISA insulin assay

INS-1E cells were seeded at an initial density of 5.0x10^5^ cells/well in each well of a 24-well plate as described in the preceding section. Following 90 min of glucose stimulation, in parallel to HTRF measurements, 20 μL of supernatant was transferred to a 96-well plate and insulin levels were measured using the insulin rodent ELISA chemiluminescence kit [American Laboratory Products Company (ALPCO), Salem, NH] as described previously [33,34]. Briefly, the ELISA assay conducted was a sandwich type immunoassay using a 96-well microplate coated with an anti-insulin monoclonal antibody. Samples and insulin standard controls were added to the plate, incubated for 2 h at room temperature with shaking and followed by 6 washes. Chemiluminescent signal was subsequently read on a PHERAstar FS microplate reader using a 1 s integration time.

### Cell viability assay

Effects of glucose stimulation on cell viability were determined using the VivaFix Cell Viability Assay (Bio-Rad Laboratories, Inc., Hercules, CA) that relies on fluorescent VivaFix dye that is concentrated into dead cells while excluded from live ones. INS-1E cells were seeded at an initial density of 5.0x10^5^ cells/well in a 24-well plate. RPMI 1640 media was exchanged 24 h after cell seeding and experiments were conducted the following day. We stimulated cells with 20 mM glucose (90 min, 37°C) while unstimulated control cells remained in KRB buffer (0 mM glucose, 90 min, 37°C) according to conditions identical to our HTRF insulin assay. Cells from each respective condition were collected with Enzyme Free Cell Dissociation Solution (EMD Millipore, Billerica, MA) and resuspended in Dulbecco’s PBS buffer (DPBS; Thermo Fisher Scientific Inc., Waltham, MA). Cells were incubated with VivaFix dye (30 min, 37°C), washed twice with DPBS, and placed into a flow cytometer (BD Accuri C6 Flow Cytometer; BD Biosciences, Franklin Lakes, NJ). Labeled and unlabeled cells in both stimulated versus unstimulated conditions were then counted by plotting the differences in fluorescence intensity measured at 675 ± 12 nm using BD CSampler Software (BD Biosciences).

### *Z´*-factor analysis

Z*′*-factor values for the HTRF insulin assay were calculated from multiple samples in a single experiment as described in Zhang (1999) [35]. We determined these values by comparing HTRF readings from different insulin concentrations (1 ng/mL versus 10 ng/mL or 2.5 ng/mL versus 10 ng/mL). Briefly, we obtained HTRF ratios from multiple replicates of each of these insulin concentrations (1, 2.5 and 10 ng/mL) that were read on a given day and used these values to calculate the mean HTRF signal and standard deviations (SD) for these respective concentrations. The Z´-factor was then defined as 1-[3(SD_higher concentration_−SD_lower concentration_)/(Mean_higher concentration_−SD_lower concentration_)] or the ratio defining the separation of respective HTRF signals between any two insulin concentrations based on bounds defined by 3 SD. We also established the Z*′*-factor for our cell-based GSIS assay by comparing multiple replicates of HTRF readings from supernatant samples collected from glucose-stimulated (20 mM glucose) versus unstimulated (KRB alone with 0 mM glucose) INS-1E cells on a single experimental day. Mean secreted insulin values were obtained from unstimulated background (KRB alone condition) and stimulated (20 mM glucose) conditions.

### Statistical Analyses

SPSS (version 18.0, IBM, Armonk, NY) was used for all statistical analyses unless stated otherwise. Fitting insulin concentrations derived from HTRF and ELISA assays to a linear regression curve yielded both the coefficient of determination (R^2^) and slope indicating extent of agreement for the respective insulin values derived from the respective methods. The R^2^ value for insulin standard curves was derived via interpolation of ratiometric fluorescence readings to a second order quadratic polynomial curve. Sigmoidal dose response curves were fit via non-linear regression of Log (ligand) versus either HTRF signal or normalized % maximal insulin secretion values. All curve fittings were plotted using GraphPad Prism (version 6.0). The respective signal to noise ratios (SNR) for the HTRF and ELISA signals were calculated as a ratio of the average fluorescence intensity to the standard deviation of background fluorescence. The coefficient of variance (CV) of HTRF measurements for estimating reagent stability was calculated as a ratio of the standard deviation (σ) to the mean (μ), or σ/μ, using the HTRF ratios obtained from measurement of the 10 ng/mL human insulin standard across 19 separate experimental days over a total period of 4 months.

The respective limits of detection (LOD) for the HTRF and ELISA-based insulin assays were determined by first calculating the Limit of Blank (LOB), where LOB is the highest apparent analyte concentration expected to be measured in blank sample (0 ng/mL standard) replicates, or LOB = mean_blank_ + 1.645(SD_blank_) [36]; we used 48 blank replicates consisting of KRB buffer for the LOB calculation. We then calculated the LOD using either the HTRF ratios or ECL signal associated with the LOB and the standard deviation associated with replicates of the lowest concentration human insulin standard (0.156 ng/mL) used in our assays, based on the formula: LOD = LOB + 3(SD_low concentration insulin sample_) where the value was expressed as an HTRF ratio or ECL signal [36,37]. These respective values were then fit to an insulin standard curve to derive the final LOD insulin concentration (expressed in ng/mL). Like the LOD, the limit of quantitation (LOQ) for both HTRF and ELISA insulin detection assays was determined using the HTRF ratios or ECL signals associated with the LOB and the standard deviation associated with replicates of the lowest concentration human insulin standard (0.156 ng/mL), based on the formula: LOD = LOB + 10(SD_low concentration insulin sample_) [37]. The resulting values were fit to an insulin standard curve to derive the final LOD concentration as expressed in ng/mL. For both LOD and LOQ calculations, GraphPad 6.0 was used to determine respective standard deviation values as well the fits for the insulin standard curves (second order polynomial quadratic regression).

For analysis of drug effects on HTRF insulin assay readings from cells and islets, respective treatment conditions were compared to control conditions via univariate ANOVA (α = 0.05) followed by Bonferroni post-hoc tests to compare between-group differences using SPSS. For determination of the proportion of secreted insulin to total cellular stores and for comparisons in single islet secretion assays, we used a 2-tailed t-test to determine significance. EC_50_ and IC_50_ values were computed via a nonlinear, least-squares regression analysis using GraphPad Prism.

## Results and Discussion

**Fig 1:**
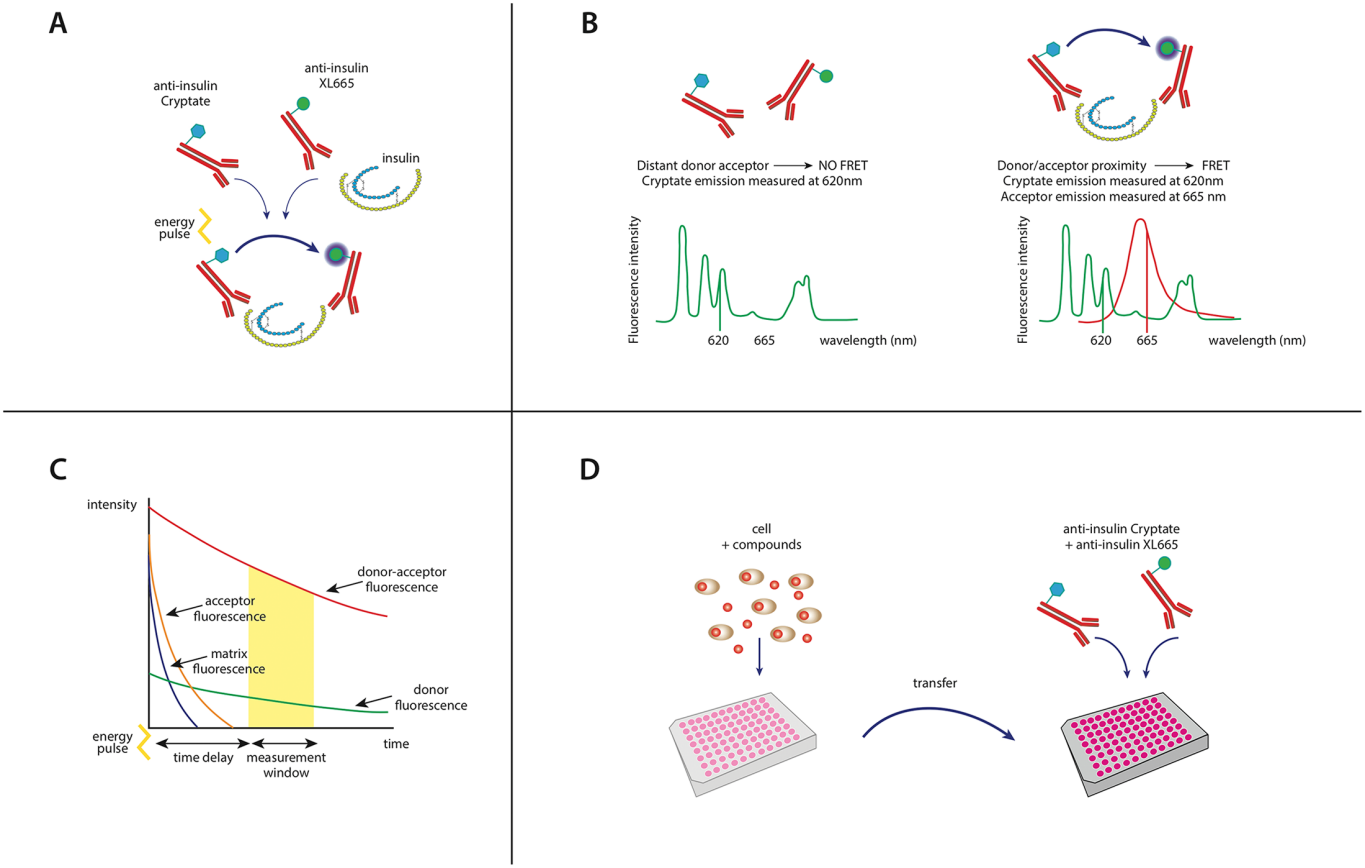
Principles of the HTRF insulin assay. **(A)** The HTRF assay is based on detection of a measurable FRET signal upon binding of anti-insulin antibodies coupled to the energy donor, Europium cryptate (EuK), and those with the near-infrared energy acceptor, XL665. **(B)** In the absence of insulin binding, there is no detectable FRET fluorescence and only donor emission is measured (λ_em_ = 620 nm) due to physical separation between the donor and acceptor fluorophores (>10 nm). When both antibodies concurrently bind insulin, the resulting physical proximity between the donor/acceptor pair results in FRET and an acceptor emission (λ_em_ = 665 nm; red emission curve). FRET is measured ratiometrically [665 nm (acceptor)/620 nm (donor)]. **(C)** Given the long-lived nature of the donor fluorescence, a 40 μs time delay prior to fluorescence measurement significantly enhances signal specificity by eliminating shorter-lived autofluorescence. **(D)** Applying these principles, we developed a homogenous insulin assay whereby levels of secreted insulin in the supernatant or within the cell are transferred to a plate where the donor and acceptor-coupled insulin antibodies are directly added, incubated and read by a plate reader.

The HTRF insulin assay we developed relies on a combination of fluorescence energy transfer (FRET) and time resolved (TR) technologies to quantify insulin levels [13,14,38]. This assay uses insulin-binding antibodies labeled with either a long-emitting energy donor, Europium cryptate (EuK), or the near-infrared energy acceptor, XL665, the emission spectrum of which is minimally contaminated by fluorescence from other compounds in solution [14] (Fig 1A). When both antibodies recognize their respective insulin epitopes, efficient and long-lived FRET occurs between the EuK donor and the XL665 acceptor (Fig 1B). We measured emission ratiometrically [665 nm (acceptor)/620 nm (donor)] to reduce well to well variation [14]. Specific detection of the stable EuK-XL665 FRET signal was significantly enhanced by the addition of a time delay that filtered out interference from transient assay buffer and protein autofluorescence (Fig 1C). We used this detection method to measure insulin released from cells or islets treated in the following manner: (1) insulin-secreting cells or islets were stimulated with secretagogue (*i*.*e*., glucose), followed immediately by (2) the transfer of the insulin-containing supernatant (or cell lysate) to a separate plate where the donor and acceptor-coupled insulin antibodies were added, incubated and measured directly without the need for additional wash steps (Fig 1D).

**Fig 2:**
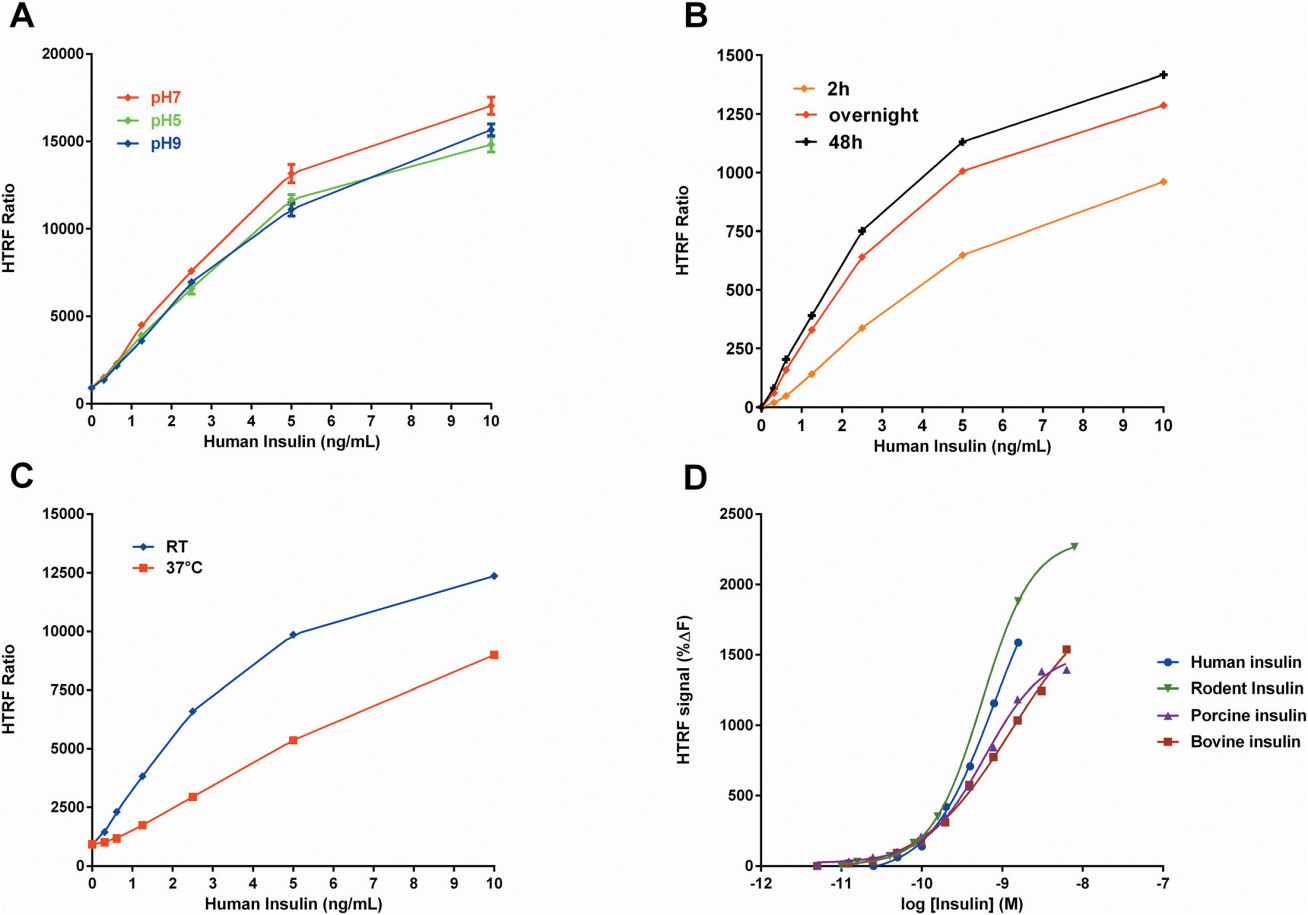
Validation of HTRF insulin assay conditions. **(A)** pH significantly affected the ratiometric HTRF signal across a range of human insulin concentrations [F(2, 40) = 21.35, p<0.001]. Antibody incubation at pH 7 yielded the greatest HTRF signal compared to other pHs (p<0.001). **(B)** 2 h antibody incubation was sufficient to produce a robust HTRF signal, with longer antibody incubation times (12 and 48 h) further increasing HTRF signal. **(C)** There was a temperature-dependent difference in HTRF values when antibodies were incubated for 2 h at room temperature (RT, 25°C) versus 37°C [F(1,29) = 16.57, p<0.001] with higher signal observed at RT. **(D)** There was no significant difference in HTRF signal between human, rodent, porcine and bovine insulin across a range of concentrations (0.01–10 nM; p>0.05). **Panels A-C:** Data are represented as the mean emitted HTRF ratio ± SEM. **Panel D:** Data are represented as %ΔF of the HTRF signal for the respective species. For all panels, the data are from experiments performed in triplicate in 384-well plates.

We first examined effects of pH, incubation time and temperature on the measurement of insulin standards, given that antibody-based detection assays in general are sensitive to these parameters [39]. Incubation at pH 7 produced higher HTRF values compared to pH 5 or 9 (p<0.001; Fig 2A), although robust signals were still detected at the pH extremes. We next assessed the change in signal strength over time by measuring HTRF values following 2, 12 and 48 h incubations. Overall, there was a progressive increase in signal strength over time (Fig 2B). Importantly, the 2 h antibody incubation produced a robust signal and a standard curve with a well-defined linear range, suggesting a reasonable balance between signal intensity to detect changes in insulin concentration and total assay time (Fig 2B). Our detection of a strong HTRF signal for at least 48 h also suggests that the assay reagents were stable throughout this period. We further examined reagent stability over a 4 month period, finding a highly reliable HTRF signal with a coefficient of variation (CV) that was only 4.3% (n = 19 trials).

We also observed a temperature-dependent difference in HTRF signal whereby antibody incubation at room temperature (25°C) produced a greater HTRF signal compared to incubation at 37°C during a 2 h incubation (p<0.001; Fig 2C). Additionally, we examined the species cross-reactivity of the insulin antibodies (Fig 2D). The antibodies recognized rodent, bovine, porcine and human insulin with no significant differences in HTRF signal (p>0.05) across a range of insulin concentrations (0.01–10 nM). Furthermore, we examined whether the insulin antibodies cross-reacted with either C-peptide or proinsulin. The antibodies did not recognize C-peptide concentrations as high as 20 μM and demonstrated ≤10% recognition of proinsulin compared to mature insulin across a low concentration range (up to100 pM) (S1 Fig). Despite a small degree of proinsulin recognition at high concentrations *in vitro*, it is likely not relevant for applications that are based on measurement of secreted insulin of which >97% is in the mature form [40]. Additionally, intracellular insulin stores are comprised almost exclusively of crystallized mature insulin with proinsulin constituting only ~10% of the total cellular insulin [40].

**Fig 3:**
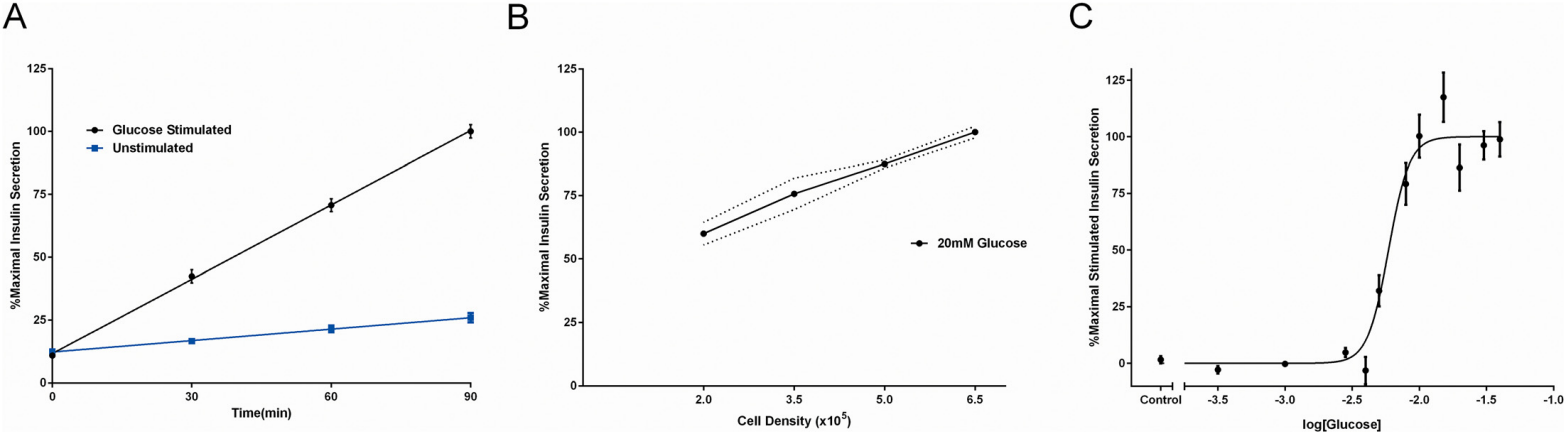
HTRF measurement of insulin secretion in INS-1E cells. **(A)** Glucose stimulation (20 mM, 37°C) significantly increased insulin secretion from pancreatic beta cell-derived INS-1E cells after 30, 60 and 90 min of treatment compared to unstimulated control (0 mM glucose; p<0.001). **(B)** Levels of secreted insulin in response to glucose stimulation (20 mM glucose, 90 min, 37°C) increased as a function of seeding cell density (R^2^ = 0.99); range of variability is indicated by the dotted lines representing SEM above and below the respective points. **(C)** Cells were stimulated with increasing concentrations of glucose (0.3–30 mM; 90 min, 37°C) and the resulting insulin secretion was fit to a sigmoidal curve (EC_50_ = 5.91 ± 0.02 mM, R^2^ = 0.85). Data are represented as % maximal insulin secretion based on mean HTRF values ± SEM from n≥3 independent experiments. HTRF measurements were performed in 96-well plates with secretion experiments performed in triplicate.

Using the parameters identified above (pH 7, 2 h incubation, 25°C), we measured insulin release from rat pancreatic beta cell-derived INS-1E cells, one of the most widely used and well-characterized cell lines currently used to study GSIS [15]. In contrast to other beta cell-derived cell lines, an important advantage of using INS-1E cells is their robust, reproducible insulin secretory response following glucose stimulation [15]. Because secretion of insulin is a dynamic process, we profiled the kinetics of release following glucose stimulation. Insulin release was significantly higher following 20 mM glucose treatment compared to unstimulated cells (KRB alone) over a 90 min time course [F(3,112) = 77.64, p<0.001; Fig 3A]. Since the glucose concentration of the culture media is 11 mM [15], we addressed the potential confound of the media’s effects on GSIS through institution of a 1 h glucose starvation step prior to our experimental manipulations (see Materials and Methods). This initial glucose starvation step has been used widely since it improves the sensitivity of insulin-secreting cells to subsequent GSIS, thus providing a greater dynamic range of secretory [41,42]. A glucose starvation step also has the additional benefit of synchronizing the cells across treatments, which ultimately reduces cell-to-cell variability in rates of GSIS [43]. To further optimize this assay, we examined the effect of cell density on GSIS. As expected, the level of insulin secretion induced by 20 mM glucose increased linearly with the number of INS-1E cells seeded per well (R^2^ = 0.99), with the least inter-experimental variability at the two highest seeding cell densities tested (Fig 3B). Consequently, we used an initial seeding density of 5.0x10^5^ cells/well for all subsequent experiments. On the day of the assay (2 days post-seeding), the final cell density was 9.5 ± 0.3x10^5^ cells/well.

We next examined the concentration-dependence of GSIS in INS-1E cells (Fig 3C) and found the EC_50_ of glucose to be 5.91 ± 0.02 mM (R^2^ = 0.85). Moreover, because previous work has shown that islets release only a fraction of their total cellular insulin stores during GSIS [44], we determined the percentage of the maximal glucose-stimulated insulin release (at 20 mM glucose stimulation) relative to the total intracellular insulin content and found it to be 2.22 ± 0.37% compared to 0.56 ± 0.06% in the unstimulated state (0 mM glucose) (p<0.001; S2 Fig).

Based on the above data, we chose a concentration of 20 mM glucose (90 min, 37°C) to elicit GSIS in our subsequent experiments. We assessed for potential glucotoxicity with a fluorescent dye-based cell viability assay in INS-1E cells (see Materials and Methods) and found no significant difference in the number of dead cells in the 20 mM glucose-stimulated group compared to the unstimulated control (p>0.05; data not shown). These data therefore strongly suggested that our glucose stimulation conditions were not cytotoxic.

**Fig 4:**
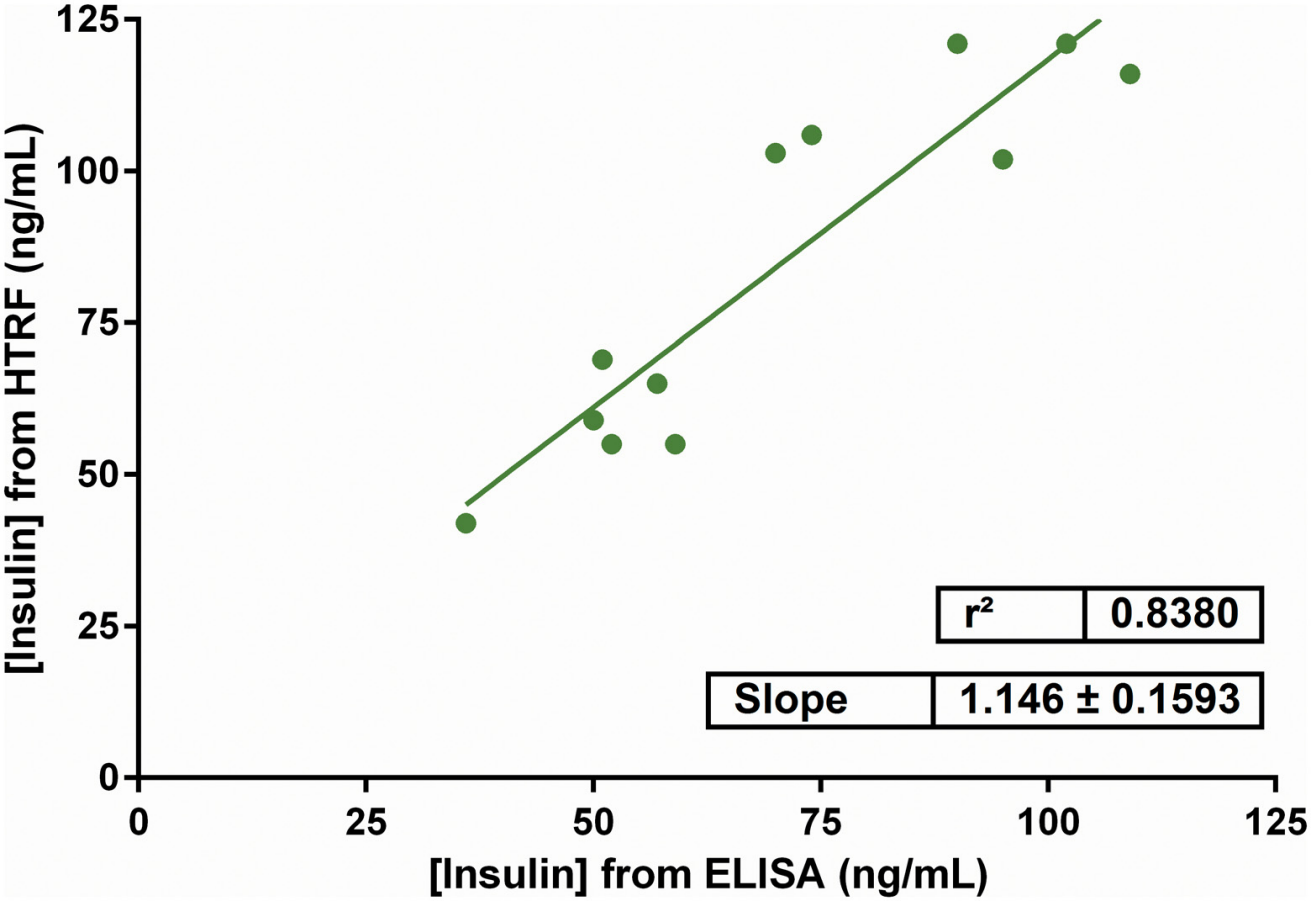
Comparison between HTRF and ELISA insulin detection assays. Supernatants collected from glucose-stimulated INS-1E cells (20 mM glucose, 90 min, 37°C) were measured concurrently with HTRF or ELISA insulin assays. The respective HTRF and ELISA assay-derived insulin concentration values were plotted. A linear regression curve of the data showed close correlation of the insulin values from the two methods (slope = 1.15 ± 0.16, R^2^ = 0.84). Results are represented as mean insulin concentrations ± SEM performed in triplicate in 3 independent experiments.

We next compared the HTRF insulin assay directly with an ELISA-based insulin assay by measuring secreted insulin from 20 mM glucose-stimulated cells from the same samples and found close correlation between the two assays (slope = 1.15 ± 0.16, R^2^ = 0.84; Fig 4). We also calculated the lower limits of detection (LOD) and quantitation (LOQ) for the HTRF assay: 0.17 ng/mL and 0.35 ng/mL of insulin, respectively (S3 Fig, **Panel A**). Though the ELISA assay had slightly lower LOD and LOQ values (0.05 and 0.13 ng/mL insulin, respectively; S3 Fig, **Panel B**), the HTRF assay had a superior signal to noise ratio (SNR) of 10.02 compared to 6.68 for ELISA–a ~50% improvement. Further, we calculated the *Z´*-factor for the HTRF assay to be 0.89 (1 versus 10 ng/mL insulin; S4 Fig, **Panel A**), demonstrating that the assay is suitable for high-throughput screening (HTS) (where the *Z´*-factor is typically ≥0.50) [35]. We also determined the *Z´*-factor for the cell-based secretion assay to be 0.64 (S4 Fig, **Panel B**), which takes into account variability of secretion intrinsic to GSIS [15] and is similar to the *Z´*-factor calculated for other fluorescence-based insulin secretory assays intended for HTS, including the recently developed luciferase-tagged insulin reporter (*Z´*-factor = 0.62) [9,45].

**Fig 5:**
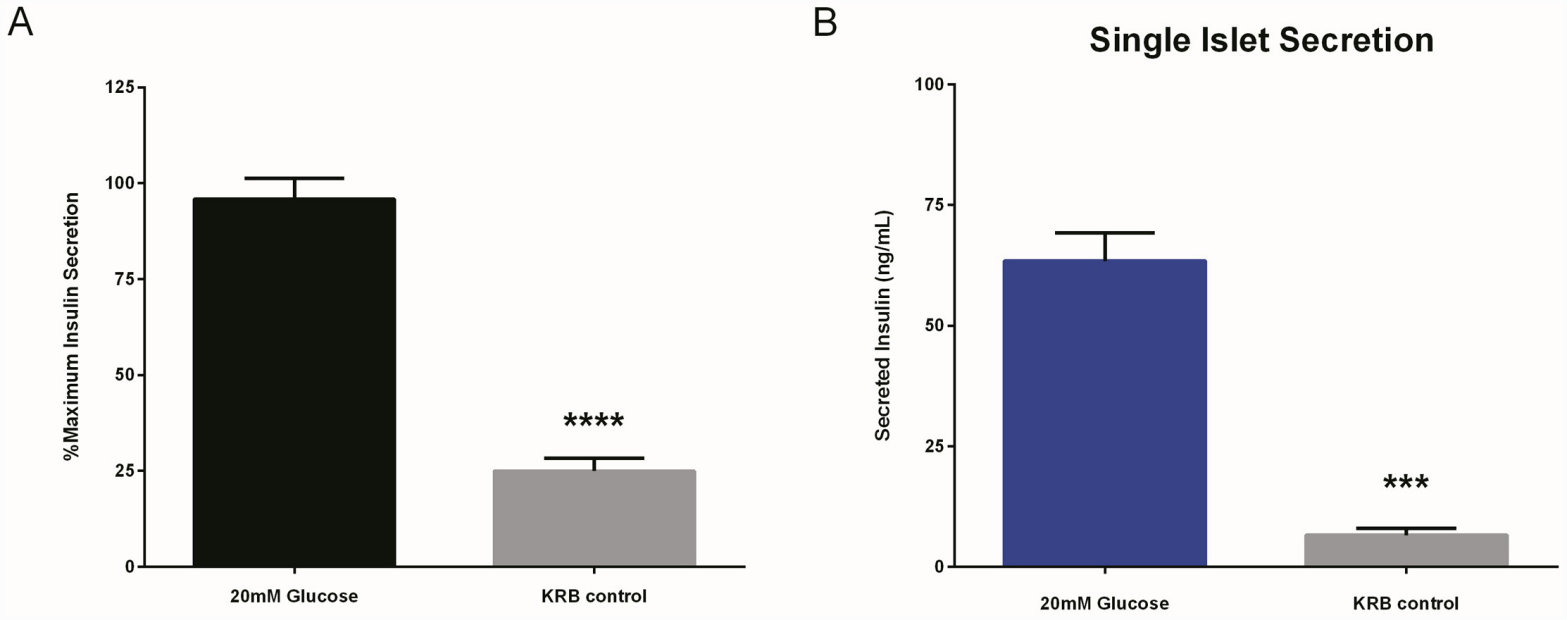
HTRF measurement of GSIS in pancreatic islets. **(A)** The HTRF insulin assay was applied to wildtype C57Bl6/J mouse-derived pancreatic islets. Islets (10/well) stimulated with 20 mM glucose demonstrated 3.8-fold stimulation of insulin secretion compared to the unstimulated control (2.8 mM glucose; p<0.001). **(B)** The HTRF insulin assay detected robust GSIS in single mouse islets, compared to unstimulated individual islets (p<0.001). Data are represented as % maximal insulin secretion (**Panel A**) or as secreted insulin concentration (ng/mL; **Panel B**) based on mean HTRF values ± SEM from n≥3 independent experiments. HTRF measurements were performed in hextuplicate in 96-well plates.

To complement the findings in INS-1E cells, we used the HTRF insulin assay to measure GSIS directly in isolated wildtype C57Bl6/J mouse-derived pancreatic islets. Similar to our findings in INS-1E cells, there was a 4-fold increase in insulin release in response to stimulation with 20 mM glucose compared to the unstimulated control (2.8 mM glucose, 90 min, 37°C; Fig 5A). We were able to detect GSIS from single islets (p<0.001; Fig 5B), underscoring the applicability of our assay to future higher-throughput applications. In addition to secreted insulin, we also measured intra-islet insulin concentrations in wildtype pancreatic islets. We found that the intra-islet insulin concentration per islet was 187.2 ng/mL, which is consistent with previously reported values [46]. Moreover, the intracellular insulin concentration per islet remained relatively constant, whereas the insulin concentration per well increased in proportion to the islet number (5 islets/well: 902.4 ± 47.3 ng/mL; 10 islets/well: 1938.7 ± 47.3 ng/mL) (S5 Fig)

**Fig 6:**
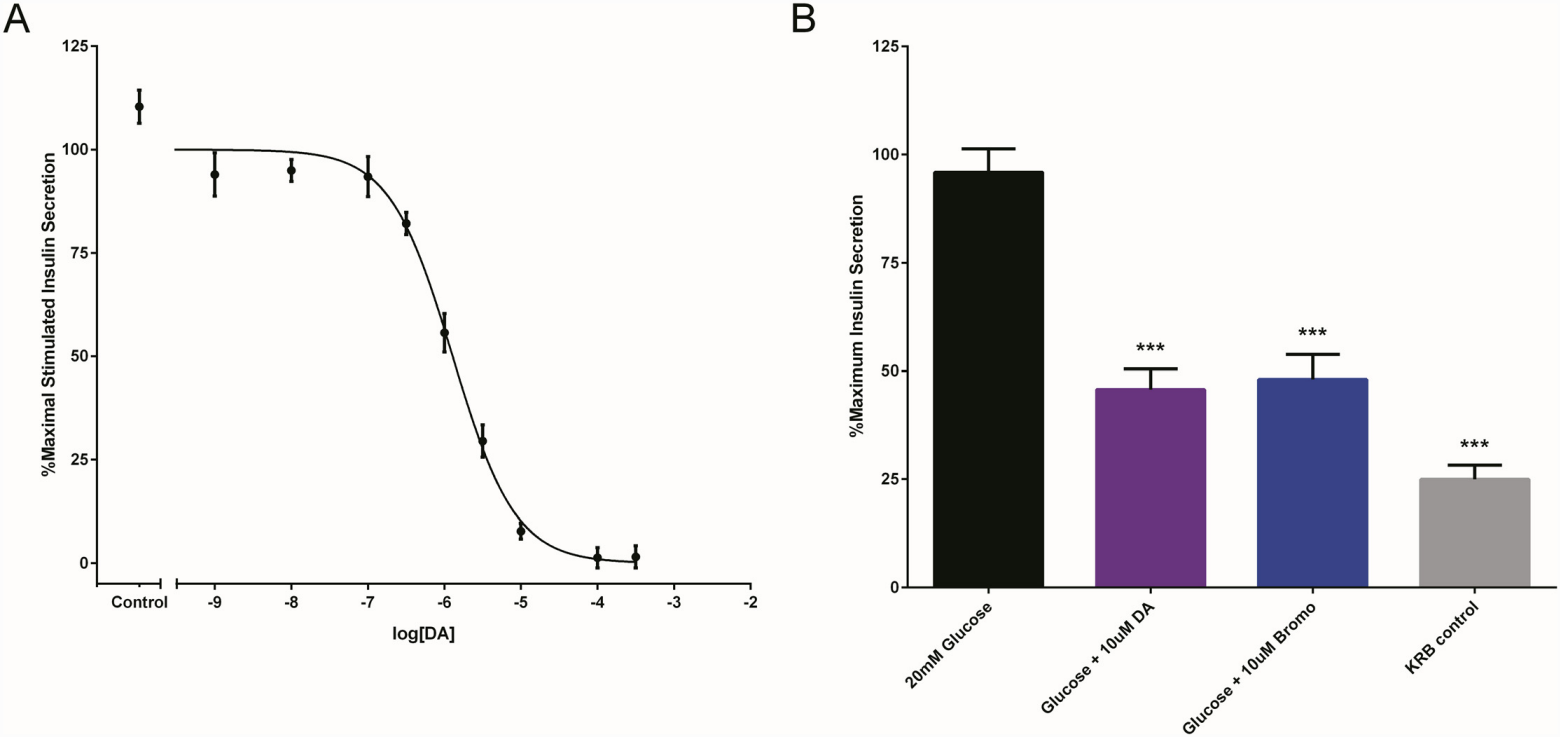
HTRF measurement of dopamine and bromocriptine effects on insulin secretion in cells and islets. **(A)** Increasing concentrations of dopamine (DA) caused dose-dependent inhibition of GSIS in INS-1E cells, which was best fit to a sigmoidal curve (IC_50_ = 1.28 ± 0.06 μM, R^2^ = 0.93). **(B)** Similarly, treatment of wildtype mouse islets with 10 μM DA significantly and comparably inhibited GSIS (p<0.001) by 70.7 ± 6.8%. Consistent with a role for dopaminergic signaling as a negative mediator of GSIS, treatment of islets with10 μM bromocriptine inhibited GSIS by 67.4 ± 8.1%. For INS-1E cell-based and mouse islet experiments (**Panels A and B**, respectively), data are represented as % maximal insulin secretion based on mean HTRF values ± SEM from n≥3 independent experiments. For all panels, HTRF measurements were performed in 96-well plates with INS-1E cell secretion experiments performed in triplicate and mouse islet experiments performed in hextuplicate.

Given recent work suggesting that DA can decrease GSIS as part of an autocrine and/or paracrine negative feedback mechanism [3,16,17], we applied the HTRF insulin assay to our cell-based system both to further elucidate the role of DA in GSIS as well as to provide additional validation of the assay in a biological context. We found that DA dose-dependently inhibited GSIS in INS-1E cells (IC_50_ = 1.28 ± 0.06 μM, R^2^ = 0.93; Fig 6A). Maximal inhibition was achieved at 10 μM DA, which fully blocked GSIS (defined as the difference between maximal stimulated and basal insulin secretion). Similarly, DA treatment (10 μM) of islets significantly inhibited GSIS by 70.7 ± 6.8% (p<0.001; Fig 6B). These data provided the requisite proof-of-principle confirmation of our assay’s ability to detect effects of dopaminergic signaling on GSIS.

Beyond validation of the HTRF assay, we used the assay to shed light on the cellular mechanisms of action of bromocriptine, which has recently been approved for improving glycemic control in type II diabetes [20,22]. Though bromocriptine lowers elevated blood glucose and insulin levels in rodent models [47–50] as well as in humans [21,51–54], the precise mechanisms by which this drug achieves these effects remain poorly understood. To date, the majority of work studying bromocriptine’s metabolic effects has focused on its actions in the central nervous system (CNS) [18]. However, a potentially important clue to elucidating the drug’s metabolic actions is its agonism of D2R and D3R [55]. Thus, given our data here, prior work demonstrating a role for DA signaling in inhibiting pancreatic islet GSIS [3,16,17], and evidence of D2R and D3R expression in insulin-secreting pancreatic islets [16], we hypothesized that bromocriptine may act directly on pancreatic islet D2R and/or D3R to modify GSIS. Consistent with this hypothesis, 10 μM bromocriptine significantly reduced GSIS by 67.4 ± 8.1% in mouse islets (p<0.001) which is a level of GSIS inhibition similar to that caused by DA (Fig 6B). These findings validate earlier reported findings [24] and suggest that, in addition to its actions in the CNS, bromocriptine can also act directly on pancreatic islets.

While we and colleagues demonstrated earlier that DA D_2_-like receptors are expressed in insulin-secreting pancreatic beta cells [3,16,17], there is recent evidence in both rat and human islets suggesting that other pancreatic islet cell types (alpha, delta and pancreatic polypeptide cells) also express DA receptors (including D2R) [56,57]. This opens the possibility that bromocriptine’s inhibition of GSIS may be due to the drug’s effects on multiple dopamine receptor-expressing islet cell types. Taken together, these data provide a framework with which to further dissect the underlying molecular mechanisms for bromocriptine’s clinically-relevant effects on insulin release [18,20,21].

## Conclusions

We have demonstrated that our HTRF-based insulin assay is a powerful tool with several advantages over the current predominantly used approaches for insulin measurement (*i*.*e*. ELISA and RIA) including the ability to rapidly and effectively measure insulin secretion with fewer steps and at less cost per sample (summarized in Table 1 and S1 Table). The HTRF insulin assay provides a platform not only for the identification of novel mechanisms regulating GSIS but also for further development of rapid HTS assays with which to identify new therapies for disorders of insulin secretion, thus significantly expanding our methodological toolbox.

**Table 1:** Advantages of the HTRF-based insulin assay compared to ELISA and RIA approaches. Here we show the main advantages of an HTRF insulin assay over comparable RIA and ELISA-based methods. Though all three methods are similarly sensitive and specific for insulin detection, the homogenous nature of the HTRF assay eliminates numerous reagents and mixing, washing and blocking steps, making the assay shorter, less expensive and more amenable to medium and high-throughput screens.

HTRF	ELISA	RIA
**Fewer steps:** Prepare fewer solutions and mix fewer reagents	**Many time-intensive steps:** Multiple incubation, blocking and washing steps	**Many time-intensive steps:** Long incubation steps and high number of reagents
**Lowest cost per sample ($0.02/sample)**	**Relatively High cost per sample (>$2/sample)**	**Medium to high cost per sample ($~1.50/sample)**
**Results within 2 h**	**Results from 4 h to overnight**	**Multiple-day results**
**Non-radioactive**	**Non-radioactive**	**Radiation exposure risk**
**Amenable to medium/high throughput screening**	**Relatively low throughput**	**Relatively low throughput**
**Homogenous assay**	**Non-homogenous assay**	**Non-homogenous assay**
**Highly sensitive and specific**	**Highly sensitive and specific**	**Highly sensitive and specific**

## Supporting Information

S1 Fig Insulin antibody recognition of mature versus immature forms of insulin. Insulin antibodies exhibited significantly greater recognition of mature human insulin (in blue) compared to human proinsulin across a range of concentrations (0–200 pM) [(F1, 5) = 14.00; p = 0.01). At concentrations up to 100 pM, there was ≤10% recognition of proinsulin compared to mature insulin. There was no recognition of human C-peptide (in green) by the insulin antibodies even at concentrations as high as 20 μM. Data are represented as %ΔF of the HTRF signal for the respective insulin species and are from experiments performed in triplicate in 384-well plates.
(TIF)

S2 Fig Secreted insulin relative to total cellular stores. The percentage of insulin secreted from INS-1E cells relative to total cellular stores increased 4-fold in response to glucose stimulation (20 mM, 90 min, 25°C) compared to the unstimulated (0 mM glucose) control (p<0.001). Results are represented as mean percentages ± SEM from 3 independent experiments performed in triplicate.
(TIF)

S3 Fig Limits of Detection and Quantitation for HTRF and ELISA insulin assays. **(A)** The limit of detection (LOD) for the HTRF insulin assay (0.165 ng/mL insulin) was derived from the sum of the mean HTRF ratiometric signals from 48 separate blank samples (0 ng/mL insulin) + 3 standard deviations from the mean. Likewise, limit of quantitation (LOQ) for the HTRF ratio (0.345 ng/mL insulin) was calculated from the mean HTRF ratio from the blank samples as above + 10 standard deviations from this mean. These values were then fit to a low concentration range insulin standard curve (0.156–1.250 ng/mL) to obtain the insulin concentrations corresponding to the HTRF assay LOD (in black) or LOQ (in red). **(B)** We calculated the LOD and LOQ for the ELISA insulin assay (0.05 and 0.13 ng/mL insulin, respectively) similarly by fitting the corresponding ECL signal to the low concentration range insulin standard curve (0.100–1.000 ng/mL),
(TIFF)

S4 Fig *Z´*-factor measurements for the HTRF insulin assay. **(A)** The *Z´*-factor for the HTRF assay was measured comparing 1 ng/mL versus 10 ng/mL insulin, which yielded a *Z´*-factor score of 0.89. Similarly, a comparison of 2.5 ng/mL versus 10 ng/mL provided a *Z´*-factor score of 0.85. Results were based on calculations from 40 data points of insulin concentrations and were originally presented as a poster [58]. **(B)** The HTRF values measured from supernatants of glucose-stimulated (20 mM glucose, 90 min, 37°C) and unstimulated (0 mM glucose, 90 min, 37°C) INS-1E cells were also used to calculate the *Z´*-factor (described in the Materials and Methods); n = 24 replicate samples for both stimulated and unstimulated conditions. Broken lines indicate 3 standard deviations from the mean of each respective group. The *Z´*-factor score of 0.64 indicates the assay’s suitability for high-throughput studies.
(TIFF)

S5 Fig HTRF measurement of intra-islet insulin. The HTRF insulin assay was used to determine the intra-islet insulin concentration using pancreatic islets from wildtype C57Bl6/J mice. Intra-islet insulin was measured from islet lysates collected from either 5 or 10 islets per well; the insulin concentration per well increased in proportion to the islet number (5 islets/well: 902.4 ± 47.3 ng/mL; 10 islets/well: 1938.7 ± 47.3 ng/mL). Data are represented as total intra-islet insulin concentration (ng/mL) based on mean HTRF values ± SEM; HTRF measurements were performed in hextuplicate in 96-well plates.
(TIFF)

S1 File NC3Rs ARRIVE Guidelines Checklist. We abided by all appropriate animal care guidelines as outlined in the ARRIVE guidelines checklist for reporting animal research.
(PDF)

S1 Table Step by step comparison of HTRF versus ELISA and RIA-based approaches to insulin detection. Comparison of HTRF, ELISA and RIA-based insulin detection approaches emphasizes the significantly reduced number of steps associated with the HTRF assay versus the predominantly used ELISA and RIA methods: 4 steps for HTRF compared to 16 and 15 steps for ELISA and RIA methods, respectively.
(PDF)
