# Supplementary figures and images for: Development of a Rapid Insulin Assay by Homogenous Time-Resolved Fluorescence
Source: PLoS One. 2016 Feb 5;11(2):e0148684. doi: 10.1371/journal.pone.0148684 (PMC4743966; doi:10.1371/journal.pone.0148684)

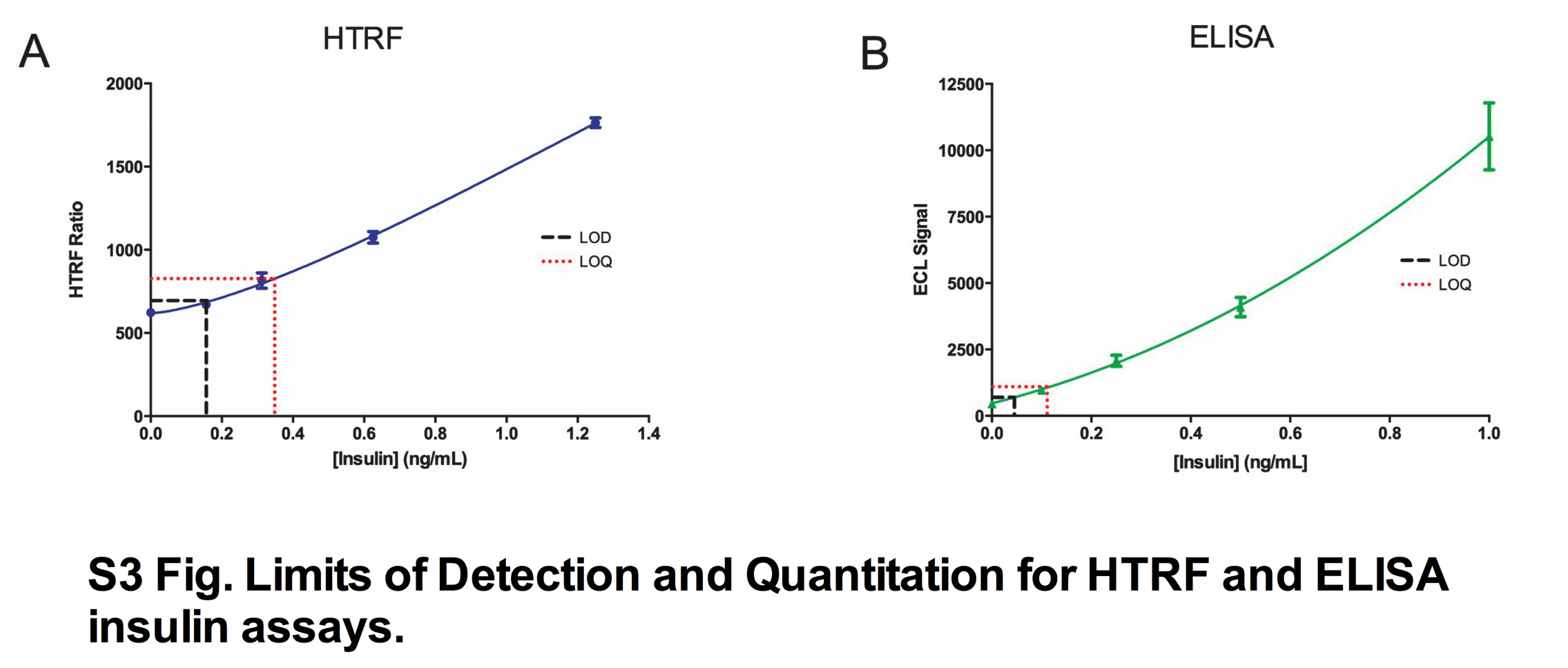

Supplement: S3 Fig — (A) The limit of detection (LOD) for the HTRF insulin assay (0.165 ng/mL insulin) was derived from the sum of the mean HTRF ratiometric signals from 48 separate blank samples (0 ng/mL insulin) + 3 standard deviations from the mean. Likewise, limit of quantitation (LOQ) for the HTRF ratio (0.345 ng/mL insulin) was calculated from the mean HTRF ratio from the blank samples as above + 10 standard deviations from this mean. These values were then fit to a low concentration range insulin standard curve (0.156–1.250 ng/mL) to obtain the insulin concentrations corresponding to the HTRF assay LOD (in black) or LOQ (in red). (B) We calculated the LOD and LOQ for the ELISA insulin assay (0.05 and 0.13 ng/mL insulin, respectively) similarly by fitting the corresponding ECL signal to the low concentration range insulin standard curve (0.100–1.000 ng/mL), (TIFF) [file pone.0148684.s003.tiff]

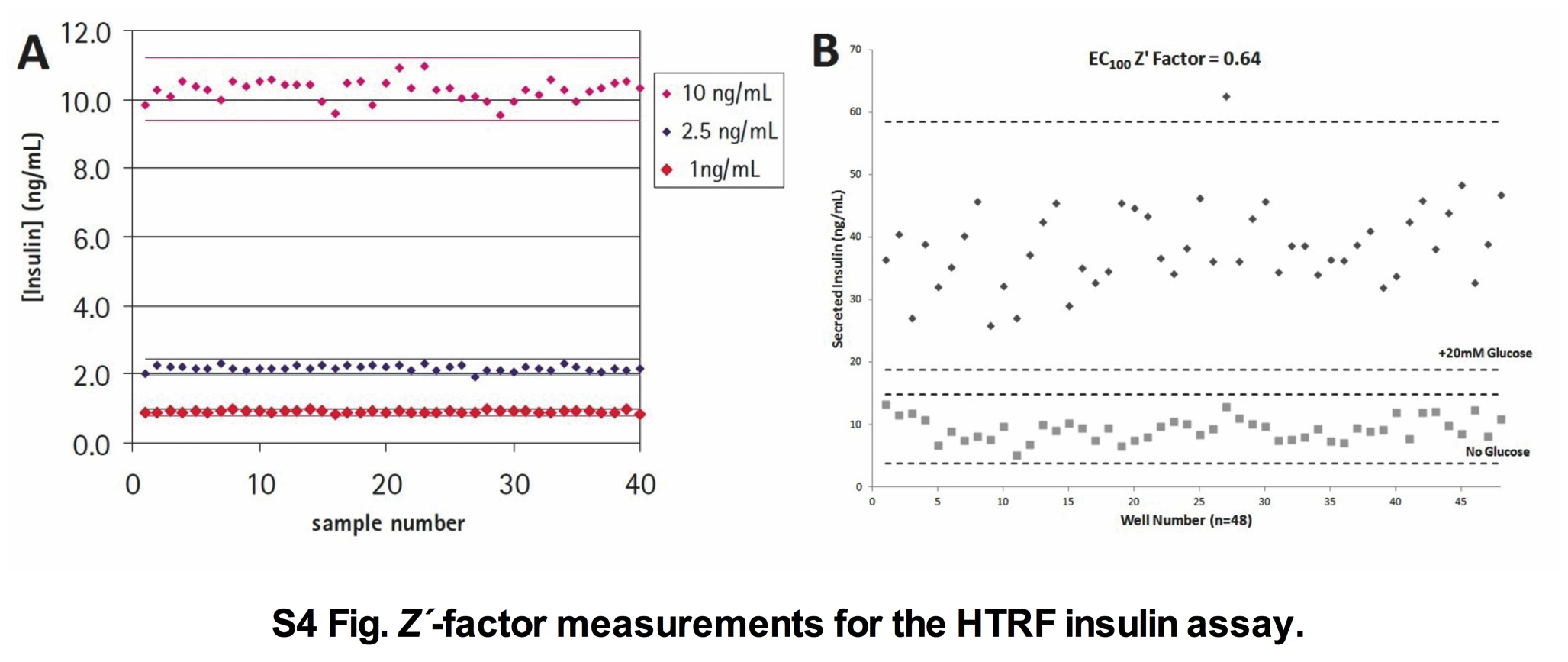

Supplement: S4 Fig — (A) The Z´-factor for the HTRF assay was measured comparing 1 ng/mL versus 10 ng/mL insulin, which yielded a Z´-factor score of 0.89. Similarly, a comparison of 2.5 ng/mL versus 10 ng/mL provided a Z´-factor score of 0.85. Results were based on calculations from 40 data points of insulin concentrations and were originally presented as a poster [58]. (B) The HTRF values measured from supernatants of glucose-stimulated (20 mM glucose, 90 min, 37°C) and unstimulated (0 mM glucose, 90 min, 37°C) INS-1E cells were also used to calculate the Z´-factor (described in the Materials and Methods); n = 24 replicate samples for both stimulated and unstimulated conditions. Broken lines indicate 3 standard deviations from the mean of each respective group. The Z´-factor score of 0.64 indicates the assay’s suitability for high-throughput studies. (TIFF) [file pone.0148684.s004.tiff]

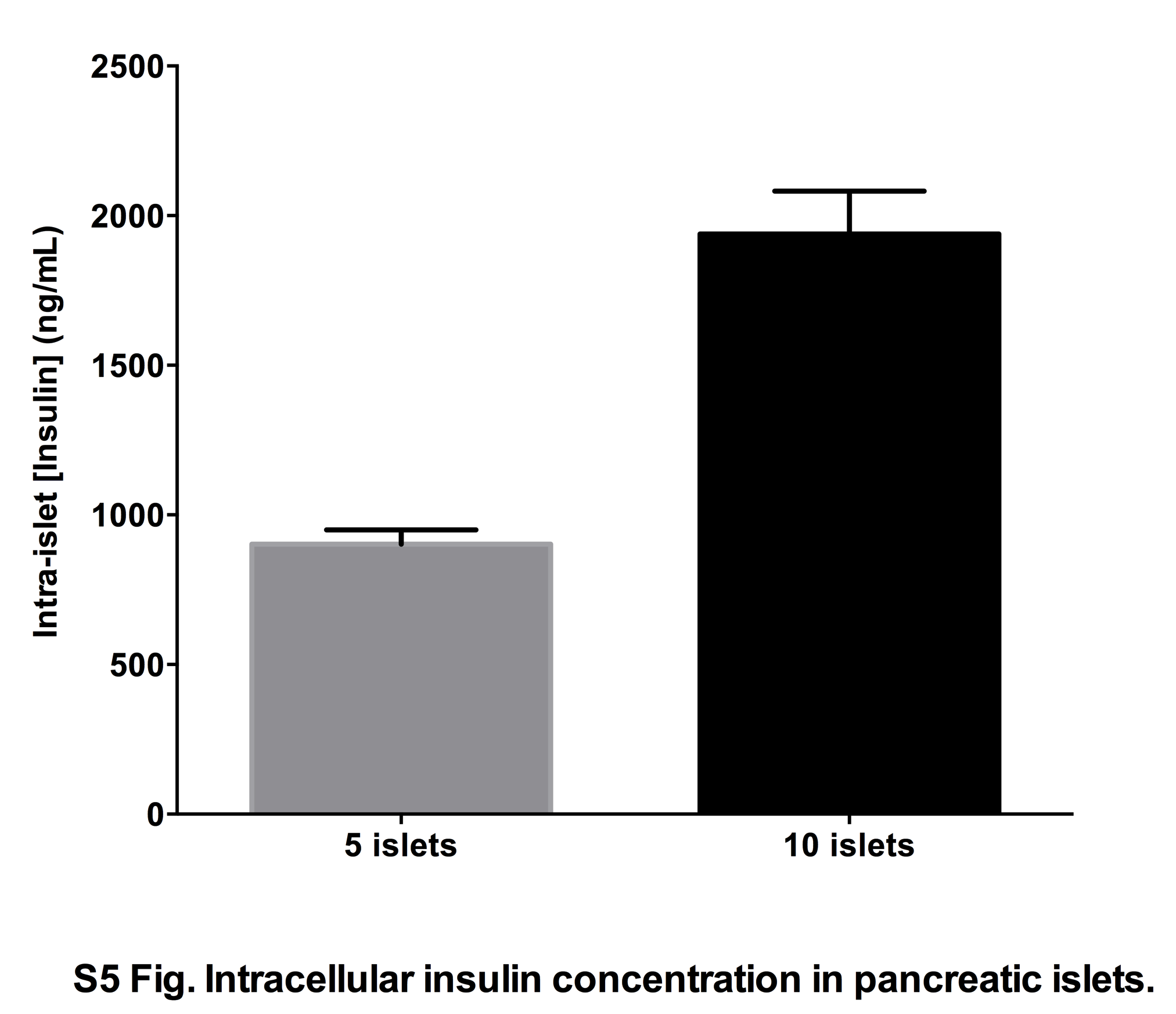

Supplement: S5 Fig — The HTRF insulin assay was used to determine the intra-islet insulin concentration using pancreatic islets from wildtype C57Bl6/J mice. Intra-islet insulin was measured from islet lysates collected from either 5 or 10 islets per well; the insulin concentration per well increased in proportion to the islet number (5 islets/well: 902.4 ± 47.3 ng/mL; 10 islets/well: 1938.7 ± 47.3 ng/mL). Data are represented as total intra-islet insulin concentration (ng/mL) based on mean HTRF values ± SEM; HTRF measurements were performed in hextuplicate in 96-well plates. (TIFF) [file pone.0148684.s005.tiff]
