# Supplementary material for: Development of a Rapid Insulin Assay by Homogenous Time-Resolved Fluorescence
Source: PLoS One. 2016 Feb 5;11(2):e0148684. doi: 10.1371/journal.pone.0148684 (PMC4743966; doi:10.1371/journal.pone.0148684)
Supplement: S1 Table — Comparison of HTRF, ELISA and RIA-based insulin detection approaches emphasizes the significantly reduced number of steps associated with the HTRF assay versus the predominantly used ELISA and RIA methods: 4 steps for HTRF compared to 16 and 15 steps for ELISA and RIA methods, respectively. (PDF) [file pone.0148684.s007.pdf]

S1 Table. Step by step comparison of HTRF versus ELISA and RIA-based approaches to insulin detection.

Step #	HTRF	Sandwich/Capture ELISA[*]	RIA[¶]
1	Add sample to plate	Apply Capture Antibody diluted in coating solution	Dilute all reagents and samples
2	Add HTRF insulin antibodies to wells with sample	Incubate 1 h at Room Temperature (25°C)	Add RIA buffer into each Nonspecific binding (NSB) tube
3	Incubate for 2+ h at Room Temperature (25°C)	Empty plate, tap out residual liquid	Add RIA buffer into each total binding (TB) tube
4	Read plate (anytime for at least 48 h)	Block plate	Set up positive controls and samples
5		Incubate for 15 min	Add first antibodies to all tubes (except NSB and TB tubes)
6		Empty plate, tap out residual liquid	Vortex, cover, and incubate at 4°C for 16-24 h
7		React sample antigens with antibodies for up to overnight	Make the stock tracer solution (STS) with RIA buffer and I^{125}-peptide
8		Empty plate, tap our residual liquid	Take a small volume and confirm concentration with γ-counter

9		Wash plate for 10 min at Room Temperature (25°C)	Make working tracer solution (WTS; 8,000-10,000 cpm/100 µL of STS into RIA buffer)
10		Repeat 3-5 times	Add 100uL WTS to each tube
11		Add secondary antibody conjugate solution	Vortex, cover and incubate at 4°C for another 16-24 h
12		Incubate for 1 h at Room Temperature (25°C)	Add second antibodies
13		Repeat wash 3-5 times	Vortex and incubate at RT for 90 min
14		React with substrate	Centrifuge to precipitate Ab-Ag complex
15		After time for color development, add stop solution into each well	Use γ-counter to measure radioactive counts per minute of pellet
16		Read with plate reader within 30 min of adding stop solution	

*ELISA protocol adapted from KPL Technical Guide (Protocols and Troubleshooting), KPL Inc., Gaithersburg, MD (2013)

¶RIA protocol taken from Phoenix Pharmaceuticals, Inc. 'General Protocol for Radioimmunoassay Kit' Burlingame, CA
